# Isolation of an accessory appendage of the left atrium using pulsed field ablation

**DOI:** 10.1093/ehjcr/ytae556

**Published:** 2024-11-13

**Authors:** Salik ur Rehman Iqbal, Christoph Gräni, Tobias Reichlin

**Affiliations:** Department of Cardiology, Inselspital, Bern University Hospital, University of Bern, Freiburgstrasse 18, 3010 Bern, Switzerland; Department of Cardiology, Inselspital, Bern University Hospital, University of Bern, Freiburgstrasse 18, 3010 Bern, Switzerland; Department of Cardiology, Inselspital, Bern University Hospital, University of Bern, Freiburgstrasse 18, 3010 Bern, Switzerland

##  

A 48-year-old man with a structurally normal heart was admitted for re-ablation of atrial fibrillation (AF) after two prior radiofrequency ablations. His first pulmonary vein isolation (PVI) was 2 years back. Later, a redo procedure with re-isolation of all PVs and isolation of the superior vena cava and cavotricuspid isthmus was performed due to recurrence. However, he continued to have episodes of AF twice weekly lasting up to 30 min.

**Figure:**
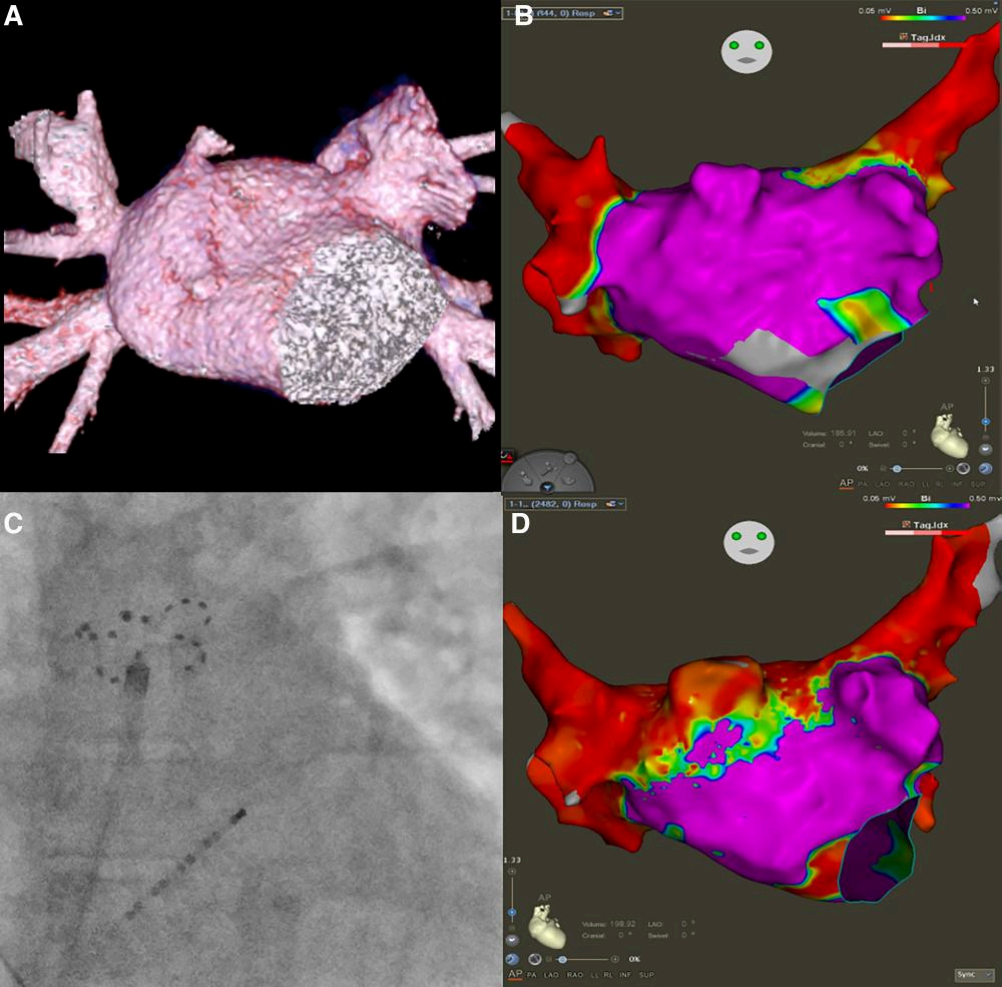


Under deep conscious sedation, a single transseptal puncture was performed. 3D electroanatomical mapping (3D-EAM) showed durable isolation of all PVs and no evidence of extra-PV low-voltage scar. An outpouching consistent with an accessory left atrial (LA) appendage (aLAA) was found on the LA anterior-superior aspect anterior to right superior pulmonary vein (RSPV) with large electrical signals and confirmed after review of a prior cardiac computed tomography scan (*Panels A* and *B*).

Empiric ablation of the roof including isolation of the aLAA was performed using pulsed field ablation (Farapulse, Boston Scientific). Repeat 3D-EAM confirmed the elimination of electrical signals from the aLAA and the roof (*Panels C* and *D*). The patient was maintained on anticoagulation and had no additional AF episodes during a follow-up of 4 months (incl. 7-day Holter after 3 months).

Accessory LAAs are cauliflower-shaped structures commonly located anterior to RSPV. Previous reports suggest their potential arrhythmogenic nature. Due to proximity to the RSPV, they can be isolated with wide area circumferential PVI. In our case, a roof line using a large footprint pulsed field ablation catheter was required to ablate the aLAA due to its distance from the RSPV (*Panels C* and *D*). While LAA isolation increases the risk of thromboembolism, it is unclear if aLAA isolation also portends the same risk and if aLAA occlusion should be considered following isolation.

Accessory left atrial appendage attached to the anterior roof of the left atrium on cardiac computed tomography scan reconstruction (*Panel A*) and 3D electroanatomical map (*Panel B*). Fluoroscopy image in anterioposterior view of the Farapulse ablation catheter covering the base of the accessory left atrial appendage at the roof of the left atrium (*Panel C*). 3D electroanatomical mapping after pulsed field ablation of the roof and accessory left atrial appendage (*Panel D*).

## Data Availability

There are no new data associated with this article.

## Notes

**Consent:** The authors confirm that informed consent for the submission and publication of this case report has been obtained from the patient.

**Funding:** No funding was obtained or required for this study.

